# Sustainable Design Approach for Modeling Bioprocesses from Laboratory toward Commercialization: Optimizing Chitosan Production

**DOI:** 10.3390/polym14010025

**Published:** 2021-12-22

**Authors:** Samir Meramo, Ángel Darío González-Delgado, Sumesh Sukumara, William Stive Fajardo, Jeffrey León-Pulido

**Affiliations:** 1 Sustainable Innovation Office, Novo Nordisk Foundation Center for Biosustainability, Technical University of Denmark, Kemitorvet 220, 2800 Lyngby-Taarbæk, Denmark; susu@biosustain.dtu.dk; 2 Nanomaterials and Computer-Aided Process Engineering, Chemical Engineering Program, Universidad de Cartagena, Piedra de Bolívar, Street 30 #48-152, Cartagena 130000, Colombia; agonzalezd1@unicartagena.edu.co; 3 Faculty of Engineering, Universidad EAN, Street 71 #9-84, Bogota 111311, Colombia; wsfajardo@universidadean.edu.co; 4 Chemical Engineering Program, Universidad EAN, Street 71 #9-84, Bogota 111311, Colombia; jleonp@universidadean.edu.co

**Keywords:** bioprocess modeling, sustainability assessment, process optimization, chitosan, TRLs

## Abstract

Enhancing the biochemical supply chain towards sustainable development requires more efforts to boost technology innovation at early design phases and avoid delays in industrial biotechnology growth. Such a transformation requires a comprehensive step-wise procedure to guide bioprocess development from laboratory protocols to commercialization. This study introduces a process design framework to guide research and development (R&D) through this journey, bearing in mind the particular challenges of bioprocess modeling. The method combines sustainability assessment and process optimization based on process efficiency indicators, technical indicators, Life Cycle Assessment (LCA), and process optimization via Water Regeneration Networks (WRN). Since many bioprocesses remain at low Technology Readiness Levels (TRLs), the process simulation module was examined in detail to account for uncertainties, providing strategies for successful guidance. The sustainability assessment was performed using the geometric mean-based sustainability footprint metric. A case study based on Chitosan production from shrimp exoskeletons was evaluated to demonstrate the method’s applicability and its advantages in product optimization. An optimized scenario was generated through a WRN to improve water management, then compared with the case study. The results confirm the existence of a possible configuration with better sustainability performance for the optimized case with a sustainability footprint of 0.33, compared with the performance of the base case (1.00).

## 1. Introduction

For the past decade, bio-based chemical production has grown globally [1]. Bio-based innovations have offered potential alternatives to reduce the strong dependence on non-renewable petrochemical sources, connected with environmental burdens [2]. Moreover, the bio-based supply chain enhances the circular bioeconomy framework [3]. Research and development (R&D) prevail as the foremost tasks for new bioprocesses to become financially competitive. Economic competitiveness and scaling-up issues are persistent limitations which have caused delays in the commercialization of many bio-based products [4]. In the end, performing R&D tasks at different levels is advantageous for bioprocess technologies emerging from laboratory protocols to reach commercialization [4]. Moreover, green chemistry principles favor upgrading and tuning new technologies to orient solutions toward pollution prevention, energy efficiency design, or using renewable feedstock [5]. There is a low rate of success in new bio-based innovations, which is an indicator of the inherent challenges of commercialization [6]. Henceforth, new methodologies that shorten the gap between commercialization and research are required.

Traditional process design methodologies were formulated with a focus on the petrochemical and chemical industries [7]. However, bioprocessing involves particular features that might not be covered by current methods. The differences between bioprocesses and chemical processes rely on many aspects, for example, the reaction mechanisms (chemistry vs. biochemistry) involving microorganisms and biocatalysts in the conversion pathways [8]. In addition, purification techniques (downstream processing) can be highly specific in bioprocesses, where the traditional unit operations like absorption, stripping, or distillation might not be appropriate [9]. Furthermore, feedstock in bioprocess is inherently biomass, while in traditional chemical systems it might vary from process to process [10]. To guarantee true sustainability, selecting the best feedstock and processing routes is essential for decision-makers (e.g., first, second, and third-generation biofuels) [11].

Whereas new bio-products are being developed at laboratory scale, comparing their sustainability performance to existing materials must be prioritized. The LCA can assist in the sustainability evaluation of a novel technology [12]. Applying optimization strategies and considering scaling effects result in improved technologies. As described, there are some challenges in modeling bioprocesses that increase the difficulty of simulating these systems at an industrial scale. Some progress is reported on scaling up techniques applied to bioprocesses under the sustainable design approach [5]. Different scaling rules based on empirical information for energy equipment were proposed [13]. A detailed work on bioprocessing scaling up was presented by Ruiz-Ruiz et al. [14] for modeling B-phycoerythrin production downstream from bench scale to the pilot plant. More recently, Piccino et al. [15] presented a framework for assisting the scale-up of chemical production systems for performing LCA for immature processes, testing the method with a nanocellulose production process [16]. With the growth of the biochemical industry, more efforts have been made toward scaling bioprocesses which have focused more on empirical data/methods for specific technologies [17]. Additionally, mathematical modeling schemes and thermodynamics are crucial in comprehending bioprocesses, given the absence of reliable and generalized frameworks [18]. Previous efforts have applied computer-aided process engineering (CAPE) to model these systems [19].

A good example of emerging bio-based technology is chitosan production. This material has gained a lot of attention mainly due to its properties and applications as a mucoadhesive, anti-inflammatory, antioxidant, and antifungal, among others [20]. Yu et al. [21] described chitosan as a low-cost polymer with multiple physicochemical and biological properties for many applications in pharma, food, crop production, or biomedicine. As an emerging process, chitosan production is still under investigation; recently, studies have modeled its production process using simulation tools [22], environmental, techno-economic, and exergy assessments [23,24,25]. Zuorro et al. [26] assessed the feasibility of a pilot-scale chitosan biorefinery using TEA. Still, optimization strategies are yet to be applied in chitosan-based biorefineries for their ongoing development to reach commercialization.

A comprehensive framework that combines feedstock and raw material selection, scaling-up, bioprocessing modeling, sustainability assessment, and process improvement to advance bioprocesses within R&D phases is still missing. Thus, the goal is to introduce a new framework to optimize bioprocesses at the low- to mid-levels of maturity to boost sustainability-driven technology innovations. The presented framework includes a procedure for scaling up lab-scale processes, green chemistry principles, and sustainability assessment. A case study for the production of chitosan from shrimp exoskeletons is applied to test the proposed method and illustrate the new possibilities of using this new framework. It will guide the R&D of upcoming bio-based projects and initiatives, including tasks to model, assess, and optimize bioprocesses. In addition to the value given by the proposed framework, this work also reports the application of optimization strategies using WRN to boost the development of chitosan biorefineries.

## 2. Materials and Methods

**Figure 1:**
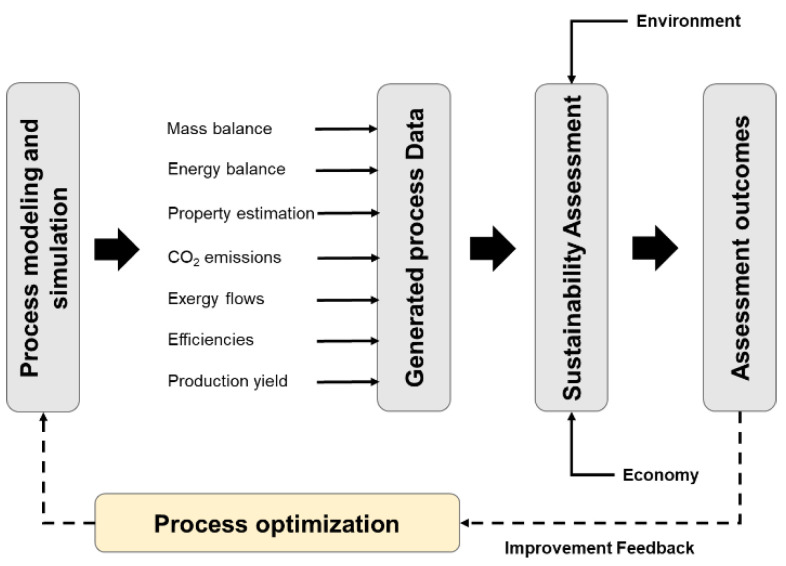
A schematic sustainability assessment approach combining process modeling, simulation, and optimization.

Coupling process modeling and simulation with assessment phases is essential to aid emerging technologies reaching higher levels of maturity. Hence, a unified framework is necessary to reach the desired R&D phases of a specific process. Evaluating sustainability performance and meeting stakeholders’ expectations is meant to assist decision-making. This approach will inform if technical areas (or the overall process) require technical improvement, allowing further optimization in a feedback step. Figure 1 shows the proposed framework to connect bioprocess modeling, simulation, and sustainability assessment to support R&D in bioprocess development.

Some milestones have to be achieved in the presented methodology. The milestones include rigorous modeling and simulation, data generation, sustainability assessment, and process optimization. These are linked in a loop procedure with feedback provided by assessment outcomes. Special attention must be taken in the process simulation step by considering the bioprocess features that differ from traditional chemical processes. The following sections explain how process simulation is featured and the interconnection between sustainability assessment and process optimization.

### 2.1. General Procedure for Modeling Bioprocesses

**Figure 2:**
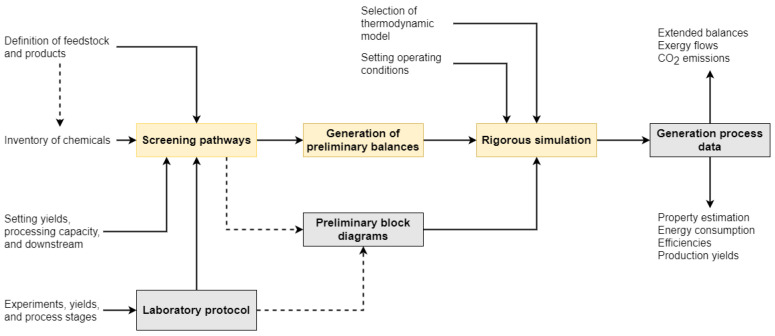
A generalized scheme to generate process data of bioprocesses and emerging technologies using process simulation.

Figure 2 displays a generalized method for modeling emerging topologies, considering the scaling-up from laboratory protocols to industrial-scale designs. The method aims to channel data for the production processes at low maturity levels to boost R&D phases to increase chances of attaining commercial success. Firstly, suitable processing pathways are screened based on available feedstock, yields, processing capacity, and potential downstream units.

The second step is the generation of preliminary mass and energy balances. Here, the scaling-up rules assist the process to new conditions (full-scale), bearing in mind its variability and uncertainties, under its current performance (lab-scale). This transitional procedure addresses the typical variations between lab-scale processes (low TRLs) and full plant designs (high TRLs). For emerging processes for which industrial data is not yet available, the method takes data from laboratory protocols, pilot-scale plants, and patents to establish the inventory of chemicals [15].

The following phase is the rigorous simulation of the process technology using CAPE. This stage requires data for process inventory, block diagrams, thermodynamic modeling, and downstream units [27]. Specific sources of this data are process simulations, industrial reports, research articles, or chemical substances databases. Finally, process simulation delivers informative data envisioning an operational, scaled-up technology, describing parameters like the extended mass and energy balances, exergy flows, or CO_2_ emissions. Process simulation also estimates thermodynamic properties, energy consumption, process efficiency, and product yield. This information is crucial for assessing a technology from a large-scale perspective, for planning, and for improving innovation early in the R&D phases.

### 2.2. A Step-Wise Framework for Simulating Bioprocesses

**Figure 3:**
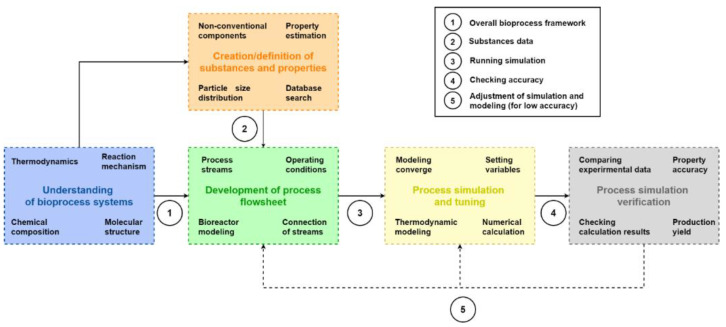
An illustration of an implemented step-wise procedure in the simulation and modeling of emerging bioprocesses.

Thanks to the advance of computers, nowadays researchers can model systems using in silico methods. In process design, it is a common practice to use process simulation software like Aspen Plus (or Super Pro Design) to model chemical process systems. In this sense, the methodology includes the use of these tools to model bioprocess systems. For the purpose of this investigation, Aspen Plus v10 (Aspen Technology, Bedford, TX, USA) was used to model the bioprocess systems. Many bioprocesses use biomass as feedstock for synthesizing products, thus involving complex chemical reactions and mechanisms (biocatalysts, microorganisms, and inhibitory effects). A comprehensive strategy for modeling and simulating bio-based topologies is presented in order to perform rigorous simulations. Figure 3 shows the procedure proposed in this study for dealing with bioprocess simulation.

The procedure acknowledges the relevance of understanding the particular characteristics of bioprocess systems, since their attributes differ, in many ways, from the traditional conception of conventional chemical processes. Reported data and laboratory experiments reports are used as data about raw materials, their molecular structures, and chemical compositions. Moreover, selecting suitable mathematical schemes and thermodynamic models that fit a system is crucial for its comprehension, given the absence of reliable and generalized frameworks [18].

The second step is the definition and creation of active substances. Process simulation tools commonly rely on a set of databases with properties of several compounds. In bioprocesses, carbohydrates, acetate, ash, and others are the primary constituent components of biomass. Many of these substances do not exist in these databases. One could use reported data on biomass processing to create those missing components [28]. The above also depends on the requirements to search available components in databases, establishing those that are not available and need to be created. It is advantageous to characterize the components and to consider their molecular structure, component type, molecular formula, and other features.

Defining biomass properties also can be performed using proximate, ultimate, and sulfate analyses [29]. This approach helps simulate thermo-biochemical processes like pyrolysis or gasification, allowing the system to be modeled based on the decomposition of elemental constituents (H_2_, O_2_, C, N, and S) under the minimization of the Gibbs free energy of formation [30]. Other aspects comprise data inclusion for particle size distribution and other physical-chemical properties depending on the selected thermodynamic model. Other models also include kinetics based approaches; extensively applied in gasification or pyrolysis processes [31]. These initial stages might embrace additional features compared to simulating well-established processes such as ethylene synthesis, methanol production, and oil refining, making the modeling and simulation of bioprocesses a differential and not-straightforward task [32].

Following the method, process flowsheets are drawn and simulation tuning are performed. Flowsheets imply interconnecting process streams (mass, energy, or work) with operating conditions and process inventory. Other features are the type of components handled in the system. There could be conventional (fluids) and non-conventional compounds. In the case of solids, particle size distribution could also be specified. Simulation tools deal with streams generally featured as mixed global streams for simulating conventional fluids associated with traditional chemical processing as a standard parameter. The following steps focus on bio-reaction modeling, downstream processing, setting operating conditions, and interconnecting mass, work, and energy streams with selected elements.

In the simulation tuning stage, the outcomes are checked to verify overall numeric convergence. If needed, some changes can be made to reach higher accuracy. This step tunes the process flowsheet by fixing the operating variable in separation systems and other units. The final step is the process simulation verification. The evaluated technologies are simulated at different TRLs. Thus, numerical verification might be challenging due to the absence of reliable data. The methodology includes a forward-backward step that provides feedback on simulation accuracy based on available data. Some sources of these data are experiments, analytical characterizations, research articles, property databases, or industrial reports. This procedure is developed from two perspectives: (i) comparison of simulated properties for a key component with experimental data, and (ii) comparison of the overall production yield compared with lab-scale or pilot plant data (low to mid TRLs) and industrial production (high TRL). Therefore, if the evaluated accuracy is unsatisfactory, the operating variables and selected models (including thermodynamics-based) must be revised and adjusted until the simulation reaches the required accuracy levels.

### 2.3. Sustainability Assessment

Sustainability assessment is a tool to strategically examine emerging and bioprocesses at different TRLs. This framework finds optimization hotspots at early R&D phases. In sustainability evaluation and process optimization, there are associated tradeoffs that must be faced [33]. As a result, making decisions toward global sustainability is not a straightforward task. Different ways to avoid ambiguous conclusions involve Pareto-based methods [34], spider diagrams [35], or aggregation for single indicators [36]. This work uses the approach employed by Sikdar et al. [37,38]. The carbon footprint is represented in Equation (1) [38].

(1) $$D={\left(\underset{n}{\prod }c_{i}\frac{y_{i}-\left(x_{i,o}-C_{offset}\right)}{x_{i}-\left(x_{i,o}-C_{offset}\right)}\right)}^{1/n}$$

D relates the aggregate sustainability metric based on index normalization and the application of geometric mean for a process alternative, $\frac{y_{i}-\left(x_{i,o}-C_{offset}\right)}{x_{i}-\left(x_{i,o}-C_{offset}\right)}$ is the normalized indicator, $c_{i}$ represents the weighting factor, and n is the number of indicators. D provides the statistical variation between assessed scenarios. This factor $\frac{y_{i}-\left(x_{i,o}-C_{offset}\right)}{x_{i}-\left(x_{i,o}-C_{offset}\right)}$ involves showing normalizing indicators as a deviation of current ($i_{j}$) and expected ($i_{j,\;worst}$) performance, divided by the difference between reference values [39].

The sustainability footprint requires setting reference values for targeting process performance. Benchmarking techniques, direct comparison, or expectations of decision-makers might be direct ways to establish these parameters [40]. This method evaluates several indicators, being a flexible and straightforward way of assessing global sustainability. Sikdar et al. [38] used the geometric mean approach to calculate a multidimensional sustainability metric, showing how far an index forms the best performance. This metric indicates how close a system is to reaching its highest sustainability performance. Lower D values reflect an improved sustainability behavior.

#### 2.3.1. Technical Indicators

A set of indicators is evaluated to determine process performance from different areas to support sustainability assessment. This characterization of indicators follows specific objectives: (i) identification of current performance, (ii) finding hotspots and bottlenecks in the system, (iii) overview of improvement areas for optimization. Table 1 summarizes the selected indicators for supporting sustainability assessment in this work. Different technical indicators are chosen to assess sustainability, including energy, process efficiency, water management, and economy [41].

Environmental sustainability is measured using the LCA methodology to analyze the production system’s performance more thoroughly [42]. Midpoint impact categories are used to link the cause–effect chain of an impact category, deliver indicators, and reflect the effects of emissions or extractions in the system. This selection is made to include more improvement areas in the sustainability assessment.

#### 2.3.2. Life Cycle Assessment

**Figure 4:**
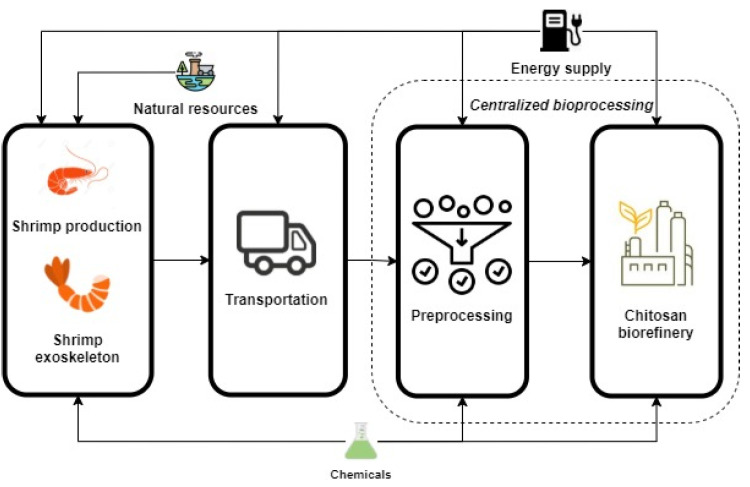
Cradle to gate boundary for chitosan production.

The LCA is directed by the ISO 14040 and ISO 14044 standards [43]. The LCA methodology involves four main stages: goal and scope, life cycle inventory modeling, impact assessment, and interpretation. The LCA was performed using the computational tool SimaPro 9.2.0.2 (PRé Sustainability, Amersfoort, The Netherlands). The ReCiPe 2016 was selected as the impact assessment, aiming to establish the environmental sustainability of the evaluated alternatives [44]. ReCiPe is a problem-oriented method for performing comprehensive impact assessment, simplifying the interpretation phase in the cause–effect chain [45]. The plant is assumed to be located in the North Colombia region, a primary shrimp production cluster. The advantage of locating the processing plant near the collection centers minimizes the impacts of logistics and transportation [46]. The LCA was evaluated through a cradle-to-gate boundary, quantifying environmental impacts over the scoped life cycle stages. This means assessing the production system from the extraction of raw materials to the factory (biorefinery) gate [47]. Figure 4 displays the life cycle stages for the production of chitosan from shrimp exoskeletons.

The goal and scope aim to assess and estimate environmental impacts for the base case and an optimized process to evaluate the improvement on the environmental sustainability performance [48]. The functional unit is set to be 1 kg of chitosan. This functional unit was selected considering the system boundaries (cradle to biorefinery gate) and the main goal of the methodology. That is, the assessment and improvement of a process technology around the factory level. Therefore, this functional unit will define the mass/energy flows and impacts in terms of chitosan.

The life cycle inventory (LCI) is a crucial phase of an LCA study. This section includes input and output data of assessed processes. The LCI was also set according to the average data collected from the Ecoinvent 3.4 database and the data generated in process simulation. The inventory consisted of input and output flows (material and energy) around the system boundaries. The data were normalized based on the functional unit. For low to mid TRLs processes, a lack of industrial-scale data is expected. Scale-up procedures and early optimization strategies are needed to screen, assess, and improve the production system [49]. The above requires connecting process modeling, sustainability assessment, and process optimization to improve new emerging technologies. In this sense, process modeling and simulation are directly linked with the LCI to generate data and run the LCA.

### 2.4. Process Optimization

The optimization of a bioprocess can be performed under different strategies with different purposes and improvement areas. This optimization makes the process more sustainable if a broader perspective is considered [50]. One cost-efficient way of reducing freshwater consumption is through water recycling and regeneration networks. Waste water streams are taken as inputs to manage water resources from outlet wastewater flows. The water integration was performed by adapting the method proposed by Kamat et al. [51], consisting of an optimization-oriented approach based on interceptor units and water regeneration. The WRN was designed considering an optimization function by minimizing freshwater and the subsequent minimization of the total treatment cost. The economic minimization included freshwater, wastewater disposal, and regeneration costs. Figure S2 (in the supplementary) shows a generic superstructure for minimizing water integration. More details about the WRN synthesis algorithm are available in the Supplementary Materials.

## 3. Case Study: Chitosan Production from Shrimp Exoskeleton

**Figure 5:**
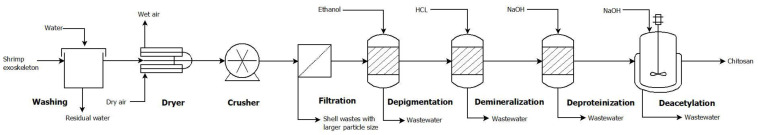
Process diagram of chitosan production from shrimp shell waste.

This section presents process modeling of chitosan production from shrimp exoskeletons. The authors in [23] presented this process. Experimental data were used to scale up and model this process topology to apply the proposed methodology. This data allowed for the establishment of the preliminary mass and energy balances, production efficiency, and the definition of interconnected units to synthesize the product. Then, we proceeded to simulate the process using Aspen Plus software. Table S1, in the Supplementary Materials, reports the inventory of chemical substances involved in this topology. This study chose the “SOLIDS” model as the thermodynamic method for property estimation. The model selection was made using the <*Aspen Plus Property Estimation subroutine*> and <*Methods Assistant option*>, indicating the type of process and substances. This model was specific for processes where solids are handled. This process comprises a pretreatment stage (washing, drying, and crushing), depigmentation, demineralization, protein rejection, and final deacetylation. In the last unit, the chitin is transformed into chitosan. Figure 5 shows the flow sheet diagram for the case study.

Shrimp shells were directed to the washing unit for cleaning, then to dried and crushed for particle size reduction. In a filter, treated shell wastes with particle-size higher than 0.5 mm were screened and extracted from the stream. The treated exoskeleton was sent to depigmentation (with ethanol) and demineralization (hydrochloric acid). These stages reject minerals and pigments (like astaxanthin) while preventing chitin hydrolysis [52]. The treated material was deproteinized via sodium hydroxide, and thus converted into chitin. This component is the primary precursor of chitosan. Partial deacetylation of chitin was developed to obtain the product using sodium hydroxide [53].

A processing capacity of 64,000 MT/y was established, taking 10% of current shrimp production for Colombia and adjacent countries that border the Pacific Ocean. Table S2 shows the chemical reactions for the primary process stages of the chitosan production process [54]. Supplementary information (Figure S1) displays the process simulation flowsheet for chitosan production from shrimp exoskeletons. Shrimp exoskeletons comprise many components, including methyl palmitate, D-N-Acetylglucosamine, and water, as summarized in Table S3. Table S4, in the Supplementary Materials, reports the material flows of the main streams generated through simulations in Aspen Plus. This process produces chitosan with a rate of 13,404 MT/y.

This case study assisted in testing the applicability of the method, considering the absence of reported industrial data and that this process remains at low/mid TRLs. The proposed strategy considers current limitations for emerging technologies to be scaled up and compared to evaluate their performance for further optimization. The method overcomes this condition by estimating the physical-chemical properties of key components in the modeled topological pathway. The method compares properties with experimental data for chitosan (main product) in the case study. Table 2 shows the detailed validation of the selected model for chitosan extraction from shrimp exoskeleton.

Table 2 indicates that molecular weight reaches high accuracy (>90%) compared them with experimental values found in the literature [55]. This simulation shows an accuracy of 98.37% for heat capacity, considering the experimental value reported by Ibrahim et al. [56]. Furthermore, study [57] developed an extraction process of chitosan from shrimp exoskeleton at the lab-scale, reaching a production yield of a 0.213 g/g shrimp exoskeleton. This simulation shows accuracy for this variable of 99%.

## 4. Sustainability Assessment

A sustainability assessment was performed for chitosan production (base case) from shrimp exoskeletons based on the data generated in the previous step (process modeling and simulation) and the sustainability footprint. This evaluation analyzed data from economic, environmental, and energy perspectives. As different areas were assessed, economic and environmental performance, and process efficiency data were needed. Two process configurations were assessed under the described assessment, bearing in mind the base case design performance and the possibility of optimizing it via WRN.

### 4.1. Base Design

The first alternative evaluated in this study was the baseline design using the configuration described in the methods section. Process simulation provides data and performance indicators for several technical areas of a production system. In this work, an improved alternative and the base design were assessed and compared. The application of green chemistry principles and water integration helped improve the system for a more sustainable design. Different metrics were selected, including energy intensity, material intensity, water consumption, improvement cost, and CO_2_ emissions. Cost evaluation was measured, considering the total treatment/improvement cost that counts the freshwater ($c_{f})$, regeneration ($c_{reg})$, and disposal of wastewater ($c_{w})$ costs, as the goal was to improve the sustainability performance of the system. These values were fixed at 1 USD/t, 0.5 USD/t, and 0.5 USD/t, respectively [51]. For the base case, the economic cost corresponded to the sum of the freshwater supply and wastewater disposal costs. Energy consumption (intensity) was also measured in this approach. The cost of energy used per treated water in water regeneration was estimated assuming these units were operating as stream strippers and a corresponding energy consumption of 7.5 × 10^−5^ kW/kg of regenerated water was considered [58].

### 4.2. Optimized Design: Including a WRN

**Figure 6:**
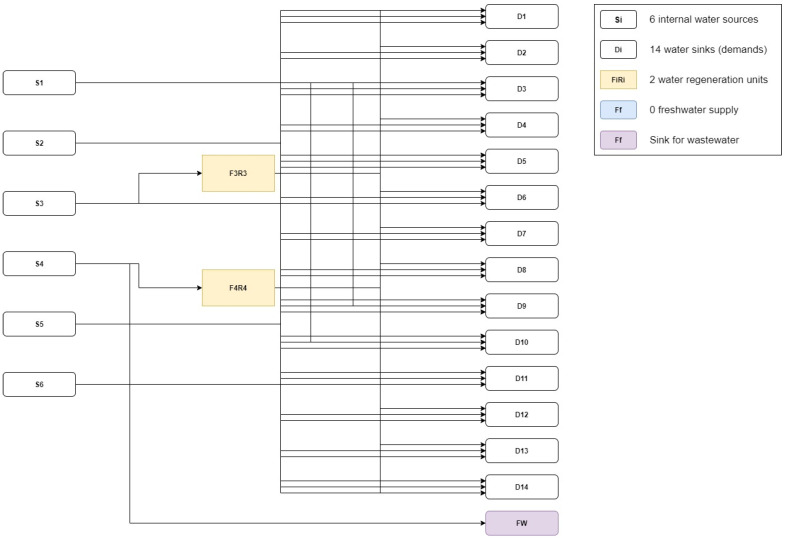
Optimized water regeneration network for the chitosan process.

Reduction in water consumption is one of the principles of green chemistry. Consequently, the first alternative for optimizing this process was to design a WRN. The installation of a WRN reduces used freshwater resources, material intensity, and offers a better environmental profile from a global perspective [59]. Wastewater, identified with $S_{i}$ $\left(S_{1},\;S_{2},\;S_{3},\;S_{4},S_{5},\;S_{6}\right)$, and freshwater, identified with $D_{i}$ ($D_{1},\;D_{2},\;D_{3},\;D_{4},D_{5},\;D_{6},\;D_{7},\;D_{8},\;D_{9},\;D_{10},\;D_{11},\;D_{12},D_{13},\;D_{14}$) were suitable for the WRN. More details of the WRN for this case are described in the Supplementary Materials. Current freshwater demand is 1,123,034.2 kg/h, which indicates that water consumption in this process is extremely high. Conversely, wastewater of the baseline design is 1,113,917.61 kg/h, which indicates a huge potential of reducing freshwater input. The minimum freshwater requirement is zero (100% reduction). Figure 6 displays the optimized WRN for the chitosan process. To design a WRN, pollutants are measured as a single group of contaminants, simplifying a single-contaminant problem. So, $C_{i}$ represents pollutants that might compromise product purity if they were to re-enter the process. Table S5 summarizes the available streams for the WRN with their corresponding mass flow, concentration of pollutants, and types of streams.

The WRN employs regeneration units to reject the pollutants in water and reduce the concentration below the required value. In order to avoid compromising the product integrity, this value was set as $C_{req}\leq 0.5\%$. If $C_{i}\leq C_{req}$, the wastewater streams can be directed to a source supply, bypassing the regeneration units. If the above condition was not met, the wastewater stream was sent to a regeneration unit and then directed to a source supply. The tradeoff of designing a WRN is that the system improves water consumption, but installing regeneration units demands an economic effort. The WRN reached the minimum freshwater target, regenerating 269,646.90 kg/h through two regeneration units and generating a total directly recycled water mass flow of 853,387.29 kg/h. The optimized design comprises six sources and fourteen demands, and employs two regeneration units to achieve water-saving objectives. Table S6 summarizes water flows and interconnecting streams for the optimized WRN.

## 5. Comparison of Alternatives

The sustainability footprint was estimated for the base and optimized designs to check the tradeoffs and performance. This allows a genuine determination of whether the improvement made in the design is worth the effort in terms of global sustainability. Table 3 summarizes the results of the evaluated indicators for both designs. As expected, there was a tradeoff between the current costs of disposal and fresh water in the water management of the plant. These results provide evidence that improving water resources through WRN might require an additional economic effort. However, the potential of water recycling in this system is vast, and the inclusion of the WRN highly improved the design’s environmental and efficiency performances. In addition to the clear tradeoff between treatment cost and water-saving metrics, energy use is another variable that might be changed by the WRN. There is a duty to operate the water regeneration units, so a higher energy consumption is obtained in the optimized design.

LCA was used to evaluate the environmental performance of the chitosan production process using a cradle-to-gate approach. Process simulations provided life cycle inventory based on the defined system. Table S7 shows the LCI for the base case and the optimized design. The Ecoinvent database was used as the primary source of background data. More details are given in the supplementary file (Table S8). The plant location was assumed to be in the Northern Colombia region (Bolivar, Colombia), where the availability of the raw material is the highest in in Colombia [24]. In order to decrease the potential environmental impacts of chitosan production derived from transportation, the plant was assumed to be a centralized facility [46].

The environmental impacts of each chitosan process alternative assessed by LCA are summarized in Table 4. Relative performances are also highlighted in Table 5, with red indicating higher impacts and blue indicating lower impacts. The contribution of the optimized process in most of the categories was slightly lower than in the base case. Although the WRN improved the overall outcome at the process boundary, the difference between both alternatives was not extraordinarily high from a life cycle perspective.

Significant differences in the resulting environmental performance were observed in the water management categories, as expected. As the optimization focused on reducing water resources, the assessment evidences an improvement in this area. In addition, the damage categories were estimated and used for the sustainability evaluation since these can be used as straightforward tools to better inform decision-makers about environmental sustainability performance. Table 5 reports the results of the damage categories for the assessed alternatives. For easier comparison, normalized impacts were used to show the environmental performance of both alternatives based on the damage categories.

**Figure 7:**
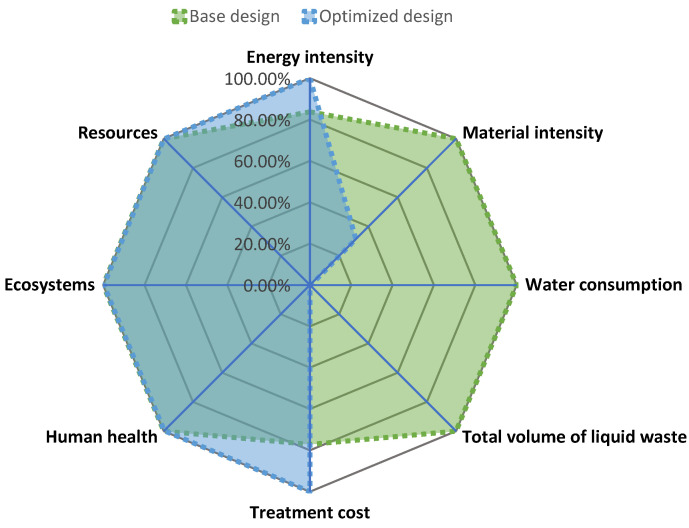
Radial chart showing normalized performance of evaluated indicators.

The sustainability footprint was then calculated to minimize the existing tradeoffs, giving a single number that embodies the overall sustainability. The technical indicators (described in Table 3) and damage categories (human toxicity, ecosystems, and resources) were used in the sustainability assessment to determine the sustainability footprint metric. Figure 7 shows a radial chart with the normalized performance of the indicators evaluated for both alternatives. In this case, the higher the normalized impact (between 0% to 100%), the lower the environmental performance. The weighting factors were set equal to unity, reflecting an egalitarian relevance of each parameter and $C_{offset}$ was fixed at 10. The resulting D for the base case was 1.0 since it is the reference parameter for comparing the alternatives, and the weighting factors were set to be 1.0. The sustainability footprint based on the geometric mean would change if the weighting factors were different to unity. Any design with higher values than the base case had a worse sustainability performance, while magnitudes below the base case represent improved performance. The optimized design showed D=0.33, indicating better a sustainability performance than the base case. This result indicates that the extra effort of installing the WRN was efficient and improved the overall sustainability performance of the process.

A second scenario was tested to analyze the variation in the sustainability footprint if the weighting factors were changed to reflect another prioritization. In this case, the analysis split the relevance grouping indicators into the following areas: energy, efficiency, water management, life cycle (environment), and economy. In this sense, water management includes indicators for measuring water-recycling performance. Each main area corresponded to a weighting factor of 1.0 (economy, energy, and efficiency), while water management and life cycle split their weighting factors between their corresponding indicators (0.5 for each water management indicator and 0.33 for each environmental impact category). The results of this scenario reflect D=0.55 for the base case and D=0.18 for the optimized case. In this scenario, the best choice based on sustainability performance was still an optimized design. These outcomes enhance the conclusion given for the first evaluated scenario, where the optimized design remains the best alternative and was genuinely an improved version of the initial design.

## 6. Conclusions

Considering sustainability in the biochemical and bioprocess industries improves the performance of new process technologies and bio-based products. Even though researchers worldwide claim that bio-based production is intrinsically sustainable, R&D must be guided to consider the challenges and tradeoffs to lead biochemical production innovations toward commercialization. Consequently, performing process modeling, sustainability assessment, and process optimization early in the design phases can accelerate biochemical production. Considering this need, a generalized method to generate process data in bioprocess systems was presented. The methodology described a particular strategy for simulating bioprocesses using CAPE. Performing integrated methods based on process simulation, sustainability assessment, and optimization would assist researchers in foreseeing future bioprocess performance to meet resource conservation and sustainability targets. The presented methodology was tested using chitosan production from shrimp exoskeletons as an emerging bioproduct with applications in many sectors.

Two process alternatives were assessed: (1) the base case and (2) an optimized case using WRN. The water regeneration network reduced water consumption in the system, minimizing the used freshwater supply. This meant a decrease of 100% in freshwater flow with a total quantity of regenerated water of 269,646.90 kg/h using two regeneration units and a total directly recycled water mass flow of 853,387.29 kg/h. The optimization was complemented with a sustainability assessment that included LCA and the evaluation of efficiency indicators and water integration costs. The results showed that the optimized design had better performance in water management and process efficiency from a multidimensional perspective. Conversely, the costs were moderately higher in this alternative due to the installation of the WRN. The optimized design was a better alternative with lower impacts and higher performance based on the sustainability footprint results. For future works, coupling the quantitative sustainability, process modeling, and optimization with data-driven methodologies might be an innovative way to introduce novelty in R&D phases, while improving the sustainability performance of emerging bioprocesses. Additionally, novel methodologies could be extended to the next level to address uncertainties, reducing the lack of data and increasing the accuracy of bio-based production systems.

## Figures and Tables

**Table 1:** Set of technical indicators for assisting sustainability assessment.

Indicator	Formula	Area	Description
Energy intensity (kW/kg p.)	$E_{i}=\frac{Energy\;consumed}{kg\;of\;product}$	Energy	The amount of energy needed for production
Material intensity (kg/kg p.)	$M_{i}=\frac{Total\;input\;mass}{kg\;of\;product}$	Process efficiency	The total input material for producing the product
Water consumption (m^3^/kg p.)	$W_{c}=Water\;consumed$	Water management	The total water consumed in production
Total liquid waste (m^3^/kg p.)	$W_{w}=liquid\;waste\;flow$	Water management	Total liquid waste flows in production
Treatment cost ($USD/kg p.)	$C_{fw}+C_{ff}+C_{rg}$	Economy	Total cost of water management in production

**Table 2:** Physical-chemical properties of chitosan provided by Aspen Plus^®^ modeling.

Property	Unit	Simulation	Experimental	Accuracy (%)
Molecular weight	g/mol	322.32	310.00	96.17
Heat capacity	Cal/mol K	132.80	135.00	98.37
Production yield	kg/kg	0.21	0.212	98.59

**Table 3:** Summary of technical indicators for design alternatives.

Process Alternative	Energy Intensity (kW/kg p.)	Material Intensity (kg/h/kg p.)	Water Consumption (kg/h/kg p.)	Total Volume of Liquid Waste (m^3^/kg p.)	Treatment Cost (USD/kg p.)
Base design	107.78	1175.05	809.60	805.88	704.60
Optimized design	128.71	369.17	3.72	0.00	913.82

**Table 4:** Environmental impacts of chitosan process alternatives based on ReCiPe End-point 2016.

Impact Category	Unit	Base Case	Optimized Case
Global warming, Human health	DALY	7.13 × 10^−4^	7.12 × 10^−4^
Global warming, Terrestrial ecosystems	species.yr	1.43 × 10^−6^	1.42 × 10^−6^
Global warming, Freshwater ecosystems	species.yr	3.89 × 10^−11^	3.88 × 10^−11^
Stratospheric ozone depletion	DALY	6.41 × 10^−7^	6.41 × 10^−7^
Ionizing radiation	DALY	6.63 × 10^−8^	6.62 × 10^−8^
Ozone formation, Human health	DALY	1.46 × 10^−7^	1.45 × 10^−7^
Fine particulate matter formation	DALY	8.71 × 10^−5^	8.70 × 10^−5^
Ozone formation, Terrestrial ecosystems	species.yr	2.10 × 10^−8^	2.10 × 10^−8^
Terrestrial acidification	species.yr	1.17 × 10^−7^	1.1710^−7^
Freshwater eutrophication	species.yr	1.13 × 10^−8^	1.13 × 10^−8^
Marine eutrophication	species.yr	9.54 × 10^−11^	9.54 × 10^−11^
Terrestrial ecotoxicity	species.yr	2.29 × 10^−9^	2.29 × 10^−9^
Freshwater ecotoxicity	species.yr	1.92 × 10^−9^	1.91 × 10^−9^
Marine ecotoxicity	species.yr	1.31 × 10^−6^	1.31 × 10^−6^
Human carcinogenic toxicity	DALY	4.01 × 10^−4^	3.98 × 10^−4^
Human non-carcinogenic toxicity	DALY	2.32 × 10^−3^	2.31 × 10^−3^
Land use	species.yr	2.88 × 10^−7^	2.88 × 10^−7^
Mineral resource scarcity	USD2013	0.07	0.07
Fossil resource scarcity	USD2013	5.92	5.91
Water consumption, Human health	DALY	1.37 × 10^−6^	1.40 × 10^−6^
Water consumption, Terrestrial ecosystem	species.yr	1.28 × 10^−8^	1.24 × 10^−8^
Water consumption, Aquatic ecosystems	species.yr	8.17 × 10^−12^	6.97 × 10^−12^

**Table 5:** Environmental impacts of damage categories for chitosan process alternatives.

Damage Category	Unit	Base Case	Optimized Design
Human health	DALY	3.52 × 10^−3^	3.51 × 10^−3^
Ecosystems	species.yr	3.19 × 10^−6^	3.18 × 10^−6^
Resources	USD2013	5.99	5.98

## Data Availability

Not applicable.

## Supplementary Materials

The following are available online at https://www.mdpi.com/article/10.3390/polym14010025/s1. Table S1: Inventory for simulation of chitosan production from shrimp exoskeleton, Table S2: Chemical reactions of chitosan extraction from shrimp exoskeleton, Table S3: Raw material composition for chitosan production from shrimp exoskeleton, Table S4: Main mass flows of chitosan production from shrimp exoskeleton, Table S5: Stream classification for applying WRN, Table S6: Summary of interconnecting flows for the WRN in chitosan production, Table S7. LCI for base case and optimized design, Table S8: Sources of background data, Figure S1: Simulation flowsheet of chitosan production from shrimp exoskeleton, Figure S2: Generic superstructure for the water regeneration network.
