# Supplementary material for: Sustainable Design Approach for Modeling Bioprocesses from Laboratory toward Commercialization: Optimizing Chitosan Production
Source: Polymers (Basel). 2021 Dec 22;14(1):25. doi: 10.3390/polym14010025 (PMC8747652; doi:10.3390/polym14010025)
Supplement: Supplementary file 1 [file polymers-14-00025-s001.zip › polymers-1513528-supplementary.pdf]

Sustainable design approach for modeling bioprocesses from laboratory to commercialization: Optimizing chitosan production

Samir Meramo[1*], Ángel Darío González-Delgado[2], Sumesh Sukumara[3], William Fajardo-Moreno[4], Jeffrey Leon-Pulido[5]

[1]*Sustainable Innovation Office, Novo Nordisk Foundation Center for Biosustainability, Technical University of Denmark, Kemitorvet 220, 2800 Kgs. Lyngby, Denmark, S.M.*

[2]*Nanomaterials and Computer-Aided Process Engineering, Chemical Engineering Program, Universidad de Cartagena. Piedra de Bolívar. Street 30 # 48-152, Cartagena 130000, Colombia, A.G.*

[3]*Sustainable Innovation Office, Novo Nordisk Foundation Center for Biosustainability, Technical University of Denmark, Kemitorvet 220, 2800 Kgs. Lyngby, Denmark, S.S.*

[4]*Faculty of Engineering, Universidad EAN, Street 71 #9 - 84, Bogotá 111311, Colombia, W.F.*

[5]*Chemical Engineering Program, Universidad EAN, Street 71 #9 - 84, Bogotá 111311, Colombia, J.L.*

[*]*Corresponding author: samhur@biosustain.dtu.dk cellphone: +45 91 67 09 14*

Supplementary information

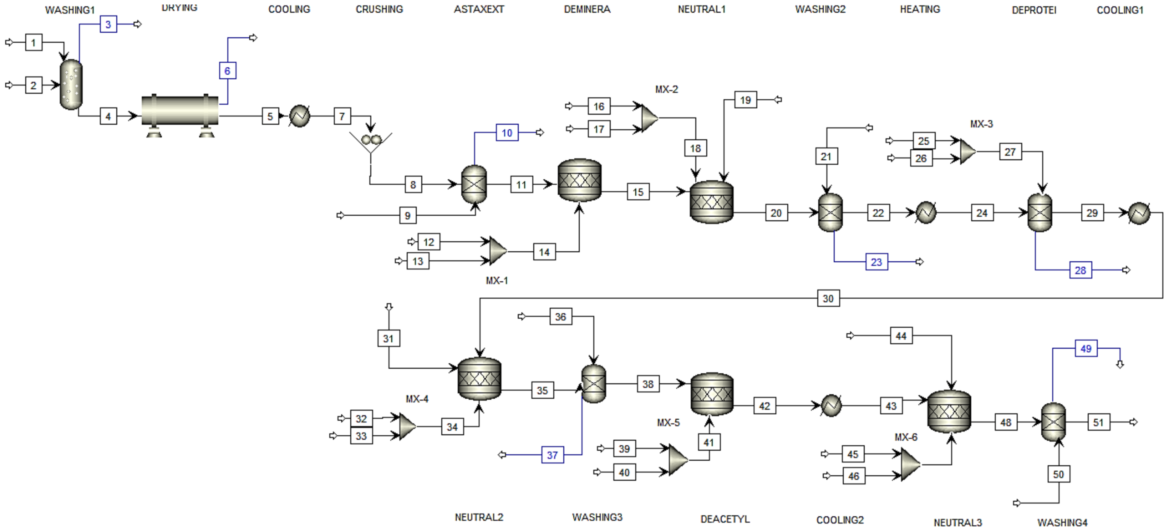

Figure S1. Simulation flowsheet of chitosan production from shrimp exoskeleton

Table S1. Inventory for simulation of chitosan production from shrimp exoskeleton

Type	Component	Available in Aspen Plus	Physical state
Feedstock-shell waste structure	Astaxanthin	✓	Solid
	Calcium carbonate	✓	Solid
	Calcium phosphate	✓	Solid

	Sodium carbonate	√	Solid
	Chitin	√	Solid
	Magnesium carbonate	√	Solid
	Methyl palmitate	√	Fluid
	L-Alanine	√	Solid
	Glutamic acid	√	Solid
	L-phenylalanine	√	Solid
	Orthophosphoric acid	√	Fluid
	Methionine	√	Solid
	Lysine	√	Solid
Solvent	Water	√	Fluid
Intermediates	Carbon dioxide	√	Fluid
Intermediates	Magnesium chloride	√	Fluid
Intermediates	Calcium chloride	√	Fluid
Solvent	Hydrogen chloride	√	Fluid
Intermediates	Cellobiose	√	Fluid
Solvent	Ethanol	√	Fluid
Reagent	Sodium hydroxide	√	Fluid
Intermediates	Sodium chloride	√	Fluid
Main product	Chitosan	√	Fluid
Intermediates	Sodium acetate	√	Fluid

Table S2. Chemical reactions of Chitosan extraction from shrimp exoskeleton

Stage	Chemical reactions
Demineralization	$CaCO_3 + 2\ HCl \rightarrow CaCl_2 + H_2O + CO_2$
	$Na_2CO_3 + 2\ HCl \rightarrow 2\ NaCl + H_2O + CO_2$
	$MgCO_3 + 2HCl \rightarrow MgCl_2 + H_2O + CO_2$
	$Ca_3(PO_4)_2 + 6\ HCl \rightarrow 3\ CaCl_2 + 2\ H_3PO_4$
Deproteinization	$C_6H_{12}N_2O_3 + 2\ NaOH \rightarrow 2\ C_3H_6NNaO_2 + H_2O$
	$C_{10}H_{16}N_2O_7 + 2\ NaOH \rightarrow 2\ C_5H_8NNaO_4 + H_2O$
	$C_{18}H_{20}N_2O_3 + 2\ NaOH \rightarrow 2\ C_9H_{10}NNaO_2 + H_2O$
	$C_{10}H_{20}N_2O_3S_2 + 2\ NaOH \rightarrow 2\ C_5H_{10}NNaO_2S + H_2O$

	$C_{12}H_{26}N_4O_3 + 2\ NaOH \rightarrow 2\ C_6H_{13}N_2NaO_2 + H_2O$
Deacetylation	$C_8H_{15}NO_6 + NaOH \rightarrow C_6H_{13}NO_5 + C_2H_3NaO_2$

Table S3. Raw material composition for Chitosan production from shrimp exoskeleton

Compound	Composition (wt.%)
Methyl palmitate	29.47
Astaxanthin	0.23
CaCO3	2.88
Ca3(PO4)2	7.23
Na2CO3	1.47
MgCO3	0.85
L-alanyl-alanine	3.62
D-N-Acetylglucosamine	16.82
Glutamic acid	6.24
Phenylalanine	2.35
Methionine	2.11
Lysine	6.82
Water	20.53

Table S4. Main mass flows of chitosan production from shrimp exoskeleton

**Stream**	**1**	**8**	**11**	**30**	**38**	**51**
Temperature (°C)	24.85	24.85	25.67	25.00	25.00	25.78
Pressure (atm)	1.00	1.00	1.00	1.00	1.00	1.00
Mass flow (t/y)	63,789	62,831.7	45,738.8	24,224.2	16,567.3	13,404.1
Methyl palmitate	0.295	0.000	0.000	0.000	0.000	0.000
Astaxanthin	0.002	0.005	0.000	0.000	0.000	0.000
Calcium carbonate	0.029	0.058	0.000	0.000	0.000	0.000
Calcium phosphate	0.072	0.145	0.000	0.000	0.000	0.000
Sodium carbonate	0.015	0.029	0.000	0.000	0.000	0.000
Magnesium carbonate	0.009	0.017	0.000	0.000	0.000	0.000
L-Alanyl-alanine	0.036	0.072	0.099	0.000	0.000	0.000
Carbon dioxide	0.000	0.000	0.000	0.000	0.000	0.000
Magnesium chloride	0.000	0.000	0.000	0.000	0.000	0.000
Calcium chloride	0.000	0.000	0.000	0.000	0.000	0.000
Hydrochloric acid	0.000	0.000	0.000	0.000	0.000	0.000
D-N-Acetylglucosamine	0.168	0.336	0.436	0.824	1.000	0.000

Glutamic acid	0.062	0.125	0.171	0.000	0.000	0.000
Phenylalanine	0.024	0.047	0.065	0.000	0.000	0.000
Orthophosphoric acid	0.000	0.000	0.000	0.000	0.000	0.000
Methionine	0.021	0.042	0.058	0.000	0.000	0.000
Lysine	0.062	0.124	0.170	0.000	0.000	0.000
Water	0.205	0.000	0.000	0.000	0.000	0.000
Ethanol	0.000	0.000	0.000	0.000	0.000	0.000
Sodium hydroxide	0.000	0.000	0.000	0.176	0.000	0.000
Sodium chloride	0.000	0.000	0.000	0.000	0.000	0.000
Chitosan	0.000	0.000	0.000	0.000	0.000	1.000
Sodium acetate	0.000	0.000	0.000	0.000	0.000	0.000

Table S5. Stream classification for applying WRN

Stream	Index	Type	Mass flow (kg/h)	C (% wt)
3	Wastewater	S1	58,621.6	0.00%
6	Wastewater	S2	6,500.12	0.00%
23	Wastewater	S3	265.100	2.23%
28	Wastewater	S4	115,531	3.83%
37	Wastewater	S5	197,557	0.52%
49	Wastewater	S6	487,435	0.31%
2	Freshwater	D1	65,024	0.01%
9	Freshwater	D2	27,713.7	0.01%
13	Freshwater	D3	53,903.4	0.01%
17	Freshwater	D4	109,514	0.01%
19	Freshwater	D5	64,029.4	0.01%
21	Freshwater	D6	23,050.7	0.01%
26	Freshwater	D7	111,355	0.01%
31	Freshwater	D8	96,132	0.01%
33	Freshwater	D9	39,656	0.01%
36	Freshwater	D10	57,679	0.01%
40	Freshwater	D11	95,112	0.01%
44	Freshwater	D12	142,071	0.01%
46	Freshwater	D13	148,441	0.01%
50	Freshwater	D14	89,353	0.01%

Table S6. Summary of interconnecting flows for the WRN in Chitosan production

Mass flows (kg/h) for demands	Mass flows (kg/h) for sources												
	Fs1	Fs2	Fs3	Fs4	Fs5	Fs6	Ffi	Fs1Ri	Fs2Ri	Fs3Ri	Fs4Ri	Fs5Ri	Fs6Ri
Fd1	0	1	7,637	0	325	29,120	0	0	0	161,826	107,821	0	0
Fd2	0	0	2,601	0	1,003	23,266	0	-	-	-	-	-	-
Fd3	0	6,300	7,162	0	325	20,825	0	-	-	-	-	-	-
Fd4	0	0	10,773	0	325	97,670	0	-	-	-	-	-	-
Fd5	0	199	6,891	0	325	31,334	0	-	-	-	-	-	-
Fd6	0	0	2,145	0	953	19,126	0	-	-	-	-	-	-
Fd7	0	0	10,956	0	325	99,329	0	-	-	-	-	-	-
Fd8	0	0	9,449	0	325	85,612	0	-	-	-	-	-	-
Fd9	18,600	0	7,432	0	325	0	0	-	-	-	-	-	-
Fd10	40,021	0	12,300	0	325	0	0	-	-	-	-	-	-
Fd11	0	0	11,949	0	325	25,437	0	-	-	-	-	-	-
Fd12	0	0	2,083	0	93,253	0	0	-	-	-	-	-	-
Fd13	0	0	2,053	0	99,097	0	0	-	-	-	-	-	-
Fd14	0	0	9,842	0	325	55,716	0	-	-	-	-	-	-
Fwi	0	0	0	7,711	0	0	-	-	-	-	-	-	-
Frid1	-	-	-	-	-	-	-	0	0	27,603	337	0	0
Frid2	-	-	-	-	-	-	-	0	0	0	844	0	0
Frid3	-	-	-	-	-	-	-	0	0	18,954	337	0	0
Frid4	-	-	-	-	-	-	-	0	0	0	746	0	0
Frid5	-	-	-	-	-	-	-	0	0	18,234	7,046	0	0
Frid6	-	-	-	-	-	-	-	0	0	0	826	0	0
Frid7	-	-	-	-	-	-	-	0	0	0	746	0	0
Frid8	-	-	-	-	-	-	-	0	0	0	746	0	0
Frid9	-	-	-	-	-	-	-	0	0	12,552	746	0	0
Frid10	-	-	-	-	-	-	-	0	0	4,287	746	0	0
Frid11	-	-	-	-	-	-	-	0	0	57,064	337	0	0
Frid12	-	-	-	-	-	-	-	0	0	0	46,735	0	0
Frid13	-	-	-	-	-	-	-	0	0	0	47,291	0	0
Frid14	-	-	-	-	-	-	-	0	0	23,132	337	0	0

Table S7. LCI for case base and optimized design

Item	Unit	Base case		Optimized design	
		Input flow	Output flow	Input flow	Output flow
Material					
Shrimp exoskeleton	kg	4.76	-	4.76	-
Water	m3	0.79	-	0.0	-
Ethanol	kg	19.98	-	19.98	-
Hydrochloric acid	kg	14.57	-	14.57	-
Sodium hydroxide	kg	9.49	-	9.49	-
Wastewater	m3	-	0.84	-	0.0055
Carbon dioxide	kg	-	0.22	-	0.22
Chitosan	kg	-	1.00	-	1.00

Utilitites					
Steam (@ 100 psi)	kg	0.14	-	0.14	-
Cooling water	kg	3.96	-	3.96	-
Electricity	kWh	0.28	-	0.33	-

Table S8. Sources of background data

**Material/energy**	**Source**
Shrimp exoskeleton	Simulated in SimaPro. Inventory is taken from [1]
Water	Tap water {CO}| tap water production, conventional treatment
Ethanol	Ethanol, without water, in 99.7% solution state, from ethylene {GLO}|
Hydrochloric acid	Hydrochloric acid, without water, in 30% solution state {RoW}| allyl chloride production, the reaction of propylene and chlorine
Sodium hydroxide	Sodium hydroxide, without water, in 50% solution state {GLO}|
Wastewater	Wastewater/m^3
Carbon dioxide	Carbon dioxide
Electricity	Electricity, medium voltage {CO}| electricity voltage transformation from high to medium voltage
Steam	Steam, in chemical industry {GLO}|
Cooling water	Tap water {CO}| tap water production, conventional treatment

Mathematical formulation for the WRN optimization model

Given a finite number of wastewater streams that belong to a water source layer (S), and these sources (s_i) are available for regeneration, recycling, or as wastewater output streams. There is a layer of water demands (D), in which these sinks (d_j) have already fulfilled their water and concentration needs. Therefore, it is desirable to synthesize a WRN considering the following aspects:

- Each source ($i = 1, 2, \dots, S$) has a flow rate (f_{s_i}) with particular pollutant concentration ($P_{s_i,v}$) for a key contaminant v.
- There are water demands ($j = 1, 2, \dots, D$) that require a particular water flow (f_{d_j}), and cannot exceed a maximum allowed concentrations of key contaminants ($C_{d_j,v}$).
- Alongside internal requirements, the WRN can include external sources in the form of freshwater (f_f) and concentration of key contaminants ($C_{f_f,v}$).

- There can be a set of wastewater streams with a defined mass flow (f_w). Also, associated regulations might restrict the outlet concentration of key contaminants ($C_{f_{w_w},v}$) in wastewater streams.
- The water regeneration is performed through some interceptor units ($FiRi$) associated with water sources. The current number of $FiRi$ units can be less than the number of sources S, since some water streams could not need regeneration. Each regeneration unit ($FiRi = F1R1, F2R2, \dots, FR$) treats a defined water flow with a particular concentration, making it available to supply process demands.
- The remaining water sources will be discharged as wastewater streams. The water integration can require external freshwater supply to meet requirements, whereas internal sources cannot meet process demands.
- Some limitations are considered for analyzing and solving the problem, and next described to understand its applicability.
- Steady-state modeling
- Aspects of the detailed engineering design of WRN are not included
- Single-pass setting for regeneration units is fixed
- There are no flow losses in regeneration units

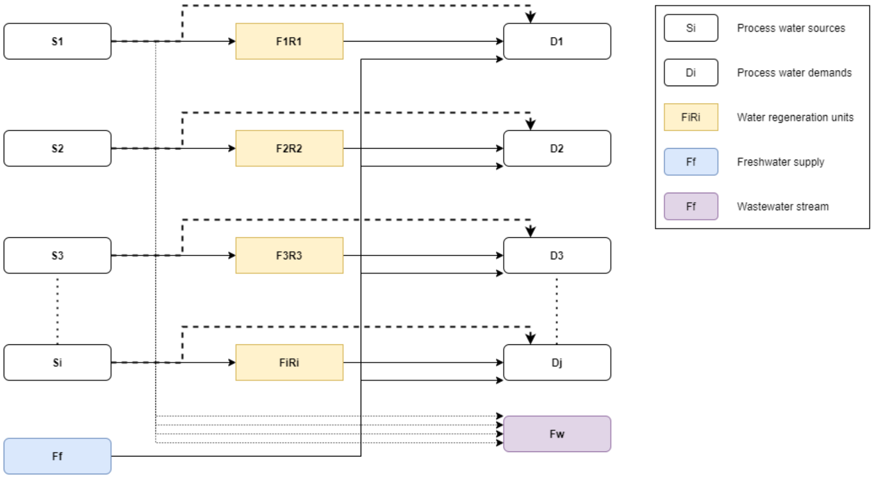

Figure S2. Generic superstructure for the Water Regeneration Network

Therefore, the task is to synthesize a WRN to optimize the water resources of a production system. The network includes mass integration via water regeneration and pollutant interceptors to minimize freshwater supply, and enhance water management within the

current design. The superstructure presented in Figure S2 is optimized using mathematical programing; this formulation can be found in the supplementary file.

Mass balance equations are used to embrace possible source/demand interconnections shown in Figure S2. The mass balance considers the flow directed from s_i to d_j, symbolized as f_{s_i,d_j}, the mass flow (f_{s_i,r_i}) that enters a regeneration unit $F_{si}r_\mathrm{i}$ and the mass flow that outs the system as wastewater (f_{s_i,w_i}). Eq. (s1) shows this relation.

$$f_{s_i} = \sum_{d_j} f_{s_i,d_j} + f_{s_i,w_i} + f_{s_i,r_i} \quad \forall s_i \quad (\mathrm{s1})$$

For process demands, the mass flow is the amount of water directed from sources s_i to a demand d_j, freshwater, and the amount of water regenerated in inceptors. Eq. (s2) shows the mass balance of process demands.

$$f_{d_j} = \sum_{s_i} f_{s_i,d_j} + f_{f,d_j} + \sum_{r_i} f_{r_i,d_j} \quad \forall d_j \quad (\mathrm{s2})$$

Water is regenerated based on the removal ratio contaminant (RR) type, representing the pollutant rejected from inlet streams. This type of unit neither uses freshwater nor generates wastewater streams. The outlet concentration ($C_{ur_i,v}$) of key contaminant v that outs a water regeneration unit, coming for an entering source flow (f_{s_i,r_i}) is given by Eq. (s3).

$$C_{ur_i,v} = \frac{\sum_{s_i} f_{s_ir_i} C_{s_i,v}(1 - RR)}{\sum_{d_j} f_{r_id_j}} \quad \forall r_i, \forall v \;\; (\mathrm{s3})$$

Each regeneration unit only can treat its corresponding process source ($s_i \rightarrow f_{si}r_i$). The total inlet and outlet flows are equal in an inceptor unit since there are no mass losses. Eq. (s4) gives the outlet concentration of contaminant v from a regeneration unit.

$$C_{ur_i,v} = C_{s_i,v}(1 - RR) \;\; \forall v \quad (\mathrm{s4})$$

As the mass balance of interceptions units, counts for inlet and outlet flows coming from sources to demands in a way that mass flows are equal between them; thus, the balance implies that $\sum_{s_i} f_{s_i,r_i} = \sum_{d_j} f_{r_i,d_j}$. The model considers the balance of key contaminants removed from water sources, as given in Eq. (s5).

$$f_{d_j} C_{ur_i,v} \geq \sum_{s_i} f_{s_i,d_j} C_{s_i,v} + f_f C_{f_f,v} + \sum_{r} f_{r_i,d_j} C_{ur_i,v} \quad \forall d_j, \forall v \quad (\mathrm{s5})$$

The minimum freshwater supply (f_{ft}) is also considered. Water targeting allows establishing process benchmarks for determining target values [2]. Eq. (s6) gives the calculation of this variable.

$$\sum_{d_j} f_f, d_j = f_{ft} \quad \forall f \quad (s6)$$

Eq. (s7) gives the formula for the total regenerated water, Eq. (s8) is the total recycled flow, and Eq. (s9) provides the total wastewater. Thus, the overall mass balance of the WRN counts the entering and leaving water flows, including internal sources, freshwater supply flows, and total wastewater flow, as given in Eq. (s9).

$$f_{rt} = \sum_{s_i} \sum_{d_j} f_{r_i d_j} \quad \forall r_i \quad (s7)$$

$$f_{sdt} = \sum_{s_i} \sum_{d_j} f_{s_i d_j} \quad (s8)$$

$$f_{wt} = \sum_{w_i} f_{w_i} \quad (s9)$$

$$f_{st} + f_{ft} = f_{wt} + f_{dt} \quad (s10)$$

f_{rt} is the total regenerated flow, f_{sdt} the direct recycled flow, f_{wt} the total wastewater flow, and f_{dt} the total demand flow. The mathematical formulation was described to synthesize a WRN considering sources splitting (or bypass), regeneration, and minimal freshwater supply. The objective function is then stated to minimize water integration cost as follows:

$$Cost = \sum_{f_{f_j}} f_{f_j}\, \delta_{f_f} + \sum_{f_{w_i}} f_{w_i} \delta_{f_w} + \sum_{r_i} \sum_{d_j} f_{r_i, d_j}\, \delta^{reg}\ \forall i\ \forall j \quad (s11)$$

$$Min\ Cost \quad (s12)$$

δ^{reg} is the cost of water regeneration. This parameter is included in the operating and capital cost for water regeneration in mass interceptors [3]. δ_{f_f} and δ_{f_w} are the cost of freshwater supply and wastewater disposal.

References

[1] Cao L, Diana JS, Keoleian GA, et al. Life cycle assessment of Chinese shrimp farming systems targeted for export and domestic sales. *Environ Sci Technol* 2011;

45: 6531–6538.

[2] Yang L, Grossmann IE. Water targeting models for simultaneous flowsheet optimization. *Ind Eng Chem Res* 2013; 52: 3209–3224.

[3] Foo D. *Process Integration for Resource Conservation*. 1st editio. Boca Raton: CRC Press. Epub ahead of print 2016. DOI: 10.1201/b12079.
